# Meis1 Regulates Epidermal Stem Cells and Is Required for Skin Tumorigenesis

**DOI:** 10.1371/journal.pone.0102111

**Published:** 2014-07-11

**Authors:** Kazuhiro Okumura, Megumi Saito, Eriko Isogai, Yoshimasa Aoto, Tsuyoshi Hachiya, Yasubumi Sakakibara, Yoshinori Katsuragi, Satoshi Hirose, Ryo Kominami, Ryo Goitsuka, Takuro Nakamura, Yuichi Wakabayashi

**Affiliations:** 1 Department of Carcinogenesis Research, Division of Experimental Animal Research, Chiba Cancer Center Research Institute, Chiba, Chiba, Japan; 2 Department of Biosciences and Informatics, Bioinfomatics Laboratory, Keio University, Yokohama, Kanagawa, Japan; 3 Department of Molecular Genetics, Graduate School of Medical and Dental Sciences, Niigata University, Niigata, Niigata, Japan; 4 Division of Development and Aging, Research Institute for Biological Science, Tokyo University of Science, Noda, Chiba, Japan; 5 Division of Carcinogenesis, Cancer Institute, Japanese Foundation for Cancer Research, Koto, Tokyo, Japan; Ohio State University Medical Center, United States of America

## Abstract

Previous studies have shown that *Meis1* plays an important role in blood development and vascular homeostasis, and can induce blood cancers, such as leukemia. However, its role in epithelia remains largely unknown. Here, we uncover two roles for *Meis1* in the epidermis: as a critical regulator of epidermal homeostasis in normal tissues and as a proto-oncogenic factor in neoplastic tissues. In normal epidermis, we show that *Meis1* is predominantly expressed in the bulge region of the hair follicles where multipotent adult stem cells reside, and that the number of these stem cells is reduced when *Meis1* is deleted in the epidermal tissue of mice. Mice with epidermal deletion of Meis1 developed significantly fewer DMBA/TPA-induced benign and malignant tumors compared with wild-type mice, suggesting that Meis1 plays a role in both tumor development and malignant progression. This is consistent with the observation that Meis1 expression increases as tumors progress from benign papillomas to malignant carcinomas. Interestingly, we found that Meis1 localization was altered to neoplasia development. Instead of being localized to the stem cell region, Meis1 is localized to more differentiated cells in tumor tissues. These findings suggest that, during the transformation from normal to neoplastic tissues, a functional switch occurs in *Meis1*.

## Introduction

Homeobox genes such as *Meis1* (myeloid ecotropic insertion site 1) are known to play a crucial role in normal development and tumor development. *Meis1* was first identified as a common viral integration site in myeloid leukemic cells of BXH-2 mice [1]. *Meis1* expression is frequently up-regulated in primary acute myeloid leukemia (AML) and acute lymphoblastic leukemia (ALL) [2]. Germline targeted knockout of *Meis1* results in embryonic lethality at day 14.5 as a result of multiple hematopoietic and vascular defects [3], [4].

*Meis1* encodes a TALE family homeodomain transcription factor that forms a heterodimeric DNA binding complex with Pbx. The interaction with Pbx1 enables Meis1 to interact with additional Hox transcription factors such as HOX-9 and HOX-10 paralog proteins. These interactions in effect functionally incorporate Meis1 into a range of Hox-dependent developmental programs [5], including vertebrate hindbrain development and limb morphogenesis [6], [7], maintenance of an undifferentiated state, and expansion of retinal progenitor cells [8], [9], and olfactory and thymic epithelial cells [10], [11].

While a number of studies have suggested that *Meis1* has a functional role in epithelial tissues, its functions in the epidermis and in skin carcinogenesis remain poorly understood. Studies of *Meis1* in epithelial tumor development have been limited to correlative studies based on gene expression and clinical outcome. As in leukemia, gene expression studies in lung adenocarcinomas [12], neuroblastomas [13], [14], [15], ovarian carcinomas [16], and nephroblastomas [17] have shown that the expression of *Meis1* is elevated in tumor tissues, suggestive of an oncogenic role. In contrast, gene expression studies in prostate cancer have shown that decreased expression of *Meis1* is correlated with poor prognosis, suggesting that it may have tumor suppression activity in prostate cancer development [18].

To gain insight into the role of *Meis1* in the epidermis, we used a tamoxifen-inducible, epithelial-specific *Meis1* knockout model in combination with a *Meis1*-EGFP reporter and cell marker co-localization studies to analyze its functions in both normal epidermal development and in skin carcinogenesis, using a two-stage carcinogenesis mouse model.

The pathology of the two-stage chemically induced skin carcinogenesis mouse model is almost identical to the development of human skin cancers and thus offers an ideal model to study skin cancer initiation and growth [19], [20]. In the first step of the chemically induced carcinogenesis protocol, mice are treated with a low dose of the mutagen 7,12-dimethylbenz(a)anthracene (DMBA) to initiate tumor development. This first chemical treatment step leads to “tumor initiation”. In the second step, mice are treated continuously with 12-*O*-tetradecanoylphorbol-13-acetate (TPA) to stimulate epidermal tumor proliferation. This second chemical treatment step influences “tumor promotion”. During tumor promotion, benign tumors, known as papillomas, are thought to arise by additional mutations caused by the TPA chemical treatment. After prolonged treatment (∼20 weeks), some of the papillomas will progress into carcinogenic tumors, such as squamous cell carcinomas (SCC). The role of various genes and cell-signaling pathways involved in skin tumor development can be explored in this two-stage skin carcinogenesis model by the use of genetically engineered mouse models [21]–[31].

In the present study, we reveal for the first time *Meis1’s* crucial function in maintaining the epidermal stem cells that act to maintain homeostasis in the epidermis. Furthermore, we present findings that demonstrate *Meis1’s* oncogenic role in epithelial tumor development. Specifically, we present findings that suggest its role in tumor development and in malignant conversion. Furthermore, our marker studies collectively indicate that *Meis1* has distinct molecular mechanisms in normal and tumorigenic tissues. Finally, we present a model for *Meis1’s* function in normal and neoplastic epidermis.

## Results

### *Meis1* is expressed in stem cells of the hair follicle bulge region in normal epidermis

**Figure 1:**
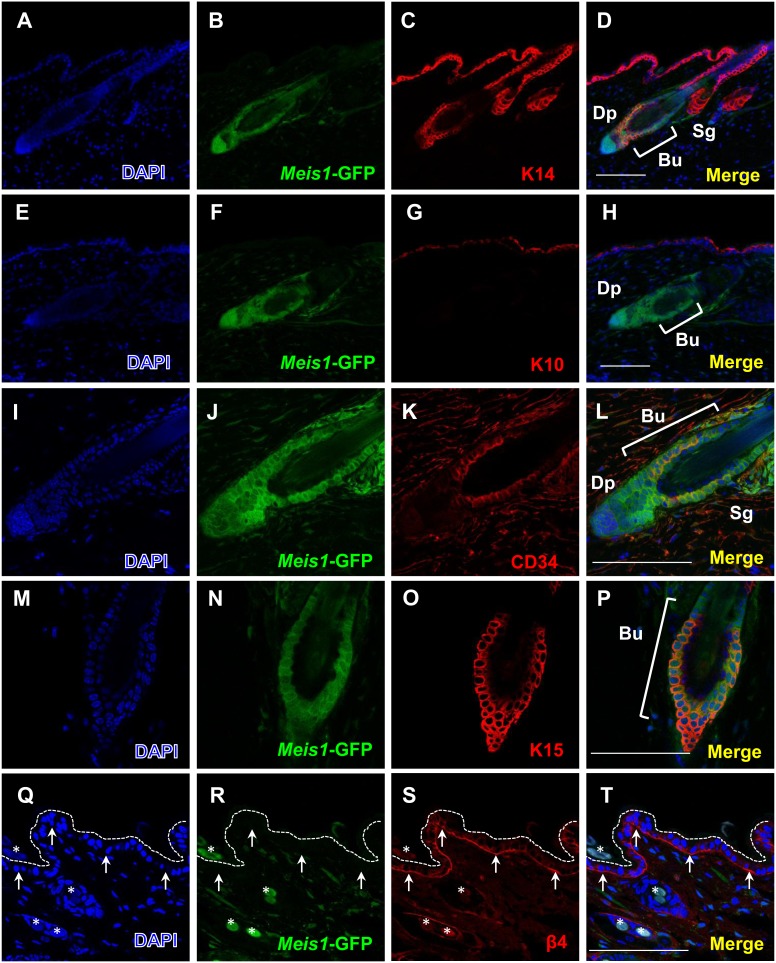
Expression of Meis1 in the bulge region including epidermal stem cells. Immunofluorescence analysis to localize *Meis1*-EGFP-positive cells in adult mouse skin. Dorsal back skin sections from 8-week-old *Meis1*-EGFP reporter mice were stained with anti-GFP antibody, in combination with anti-K14, K10, CD34, K15, and β4-integrin antibodies. Cells were counterstained with DAPI. (A–C) DAPI (blue), *Meis1*-EGFP (green) fluorescence, and K14 (red) are shown. (D) The merged image of *Meis1*-EGFP with K14. (E–G) DAPI (blue), *Meis1*-EGFP (green) fluorescence, and K10 (red) are shown. (H) The merged image of *Meis1*-EGFP with K10. (I–K) DAPI (blue), *Meis1*-EGFP (green) fluorescence, and CD34 (red) are shown. (L) The merged image of *Meis1*-EGFP with CD34. (M–O) DAPI (blue), *Meis1*-EGFP (green) fluorescence, and K15 (red) are shown. (P) The merged image of *Meis1*-EGFP with K15. (Q–S) DAPI (blue), *Meis1*-EGFP (green) fluorescence, and β4-integrin (red) are shown. (T) The merged image of *Meis1*-EGFP with β4-integrin. Arrowheads indicate the basal layer of the epidermis. Abbreviations: “Bu” means “bulge”, “Sg” means “sebaceous gland”, and “Dp” means “dermal papilla”. Scale bars, 100 µm.

The epidermis is comprised of a stratified squamous epithelium and an underlying dermis consisting of matrix-rich connective tissue. Furthermore, epidermal stem cells are found at the basal layer of the epidermis and are involved in maintaining proper epidermal architecture and function throughout an organism’s lifespan. To investigate *Meis1’s* role in the epidermis, we first examined Meis1 expression in normal skin tissue. We used a *Meis1*-*EGFP* reporter [32] in normal wild-type mice to determine where, if at all, *Meis1* is expressed in the epidermis. The *Meis1*-*EGFP* reporter was made from an artificial chromosome (BAC) transgene (RP23-306E8) corresponding to ∼80 kb upstream and ∼30 kb downstream of the mouse *Meis1* gene into which an *EGFP* cDNA was inserted just 5′ of the *Meis1* translation start site, thereby ensuring that no additional exogenous expression of *Meis1* was introduced from expression of the transgene reporter. Immunofluorescence analysis of the epidermis of 8-week-old *Meis1*-EGFP mice showed that *Meis1*-EGFP was localized to the bulge region of hair follicles (**Fig. 1A–1D**). The bulge region, where multipotent stem cells are activated upon wounding, provides progenitor cells for regeneration and repair of the epidermis or at the start of a new cycle of hair development. To investigate further *Meis1’s* role in the bulge region, we carried out co-immunostaining with *Meis1*-EGFP and epidermal stem cell markers: CD34, keratin 15 (Krt15/K15), and the basal cell marker β4-integrin. We found that *Meis1*-EGFP partially overlapped with the epidermal stem cell markers CD34 and K15 in the bulge region (**Fig. 1I–1P**) and that *Meis1*-EGFP was not co-expressed with β4-integrin in the basal layer cells of the epidermis (**Fig. 1Q–1T**).

To investigate further *Meis1* expression in the bulge region, we performed BrdU-chase experiments to label quiescent stem cells of the epidermis in *Meis1*-EGFP mice. We found that *Meis1*-EGFP signals were co-expressed with BrdU-LRCs (Label Retaining Cells) (**Fig. S2C–S2F**). We also carried out co-immunostaining with *Meis1*-EGFP and a differentiation marker, keratin 10 (Krt10/K10) (**Fig. 1E–1H**). We did not detect *Meis1*-EGFP signal overlapping with K10 in the epidermis. Taken together, these results demonstrate that *Meis1* is predominantly expressed in the epidermal stem cells of the hair follicle bulge region and suggest that *Meis1* is involved in stem cell function or maintenance in the epidermis.

### *Meis1* is required for epidermal stem cell maintenance

**Figure 2:**
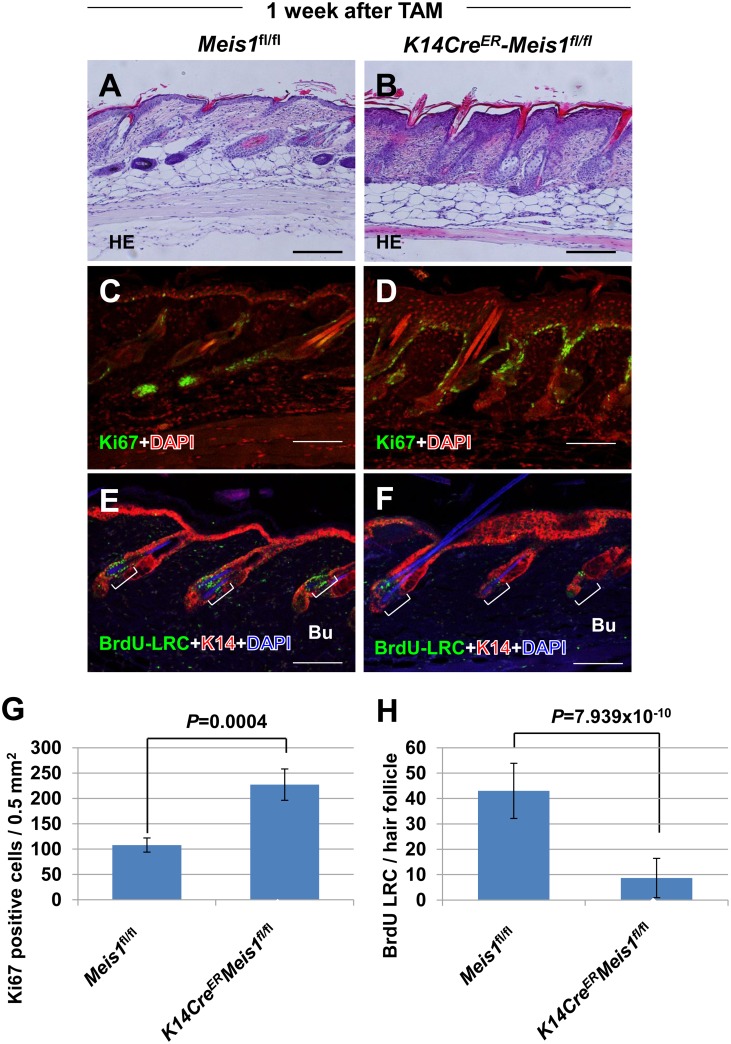
Epithelial-specific disruption of *Meis1* decreased epidermal stem cells. (A, B) Dorsal back skin HE sections of *Meis1^fl/fl^* mice (n = 3) (A) and *K14Cre^ER^-Meis1^fl/fl^* mice (n = 5) (B) one week after TAM (tamoxifen) treatment are shown. (C, D) Representative immunostaining pattern of Ki-67-positive cells (green) in the skin from control *Meis1^fl/fl^* mice (C) and *K14Cre^ER^-Meis1^fl/fl^* mice (D) one week after TAM treatment. Cells were counterstained with DAPI (red). (E, F) Representative double immunostaining pattern of BrdU-LRCs (green) and K14 (red) in the skin from control *Meis1^fl/fl^* mice (E) and *K14Cre^ER^-Meis1^fl/fl^* mice (F) one week after TAM treatment. Cells were counterstained with DAPI (blue). For chase experiments, BrdU was administered by peritoneal injection. See material and methods for more details of the BrdU chase experiments. (G) Numbers of Ki67-positive cells per 0.5 mm^2^ in dorsal back skin sections of control *Meis1^fl/fl^* (n = 3) and *K14Cre^ER^-Meis1^fl/fl^* (n = 5). Error bars are the standard deviations (S.D.). The p-value was calculated for Ki67-positive cell number by t-test. (H) Numbers of BrdU-LRCs per hair follicle in dorsal back skin sections of control *Meis1^fl/fl^* (n = 10) and *K14Cre^ER^-Meis1^fl/fl^* (n = 16). The p-value was calculated for BrdU-LRC number by *t*-test. Error bars are the standard deviations (S.D.). Abbreviation: “Bu” means “bulge”. Scale bars, 100 µm.

To investigate further the hypothesis that *Meis1* plays a role in stem cell maintenance, we generated mice harboring conditional alleles of *Meis1* (*Meis1^fl^*), in which exon 8 of the *Meis1* gene encoding the *Meis1* homeodomain (DNA-binding site) is flanked by *loxP* sites. Next, we crossed the *Meis1* ^fl^ conditional-knockout mice with mice carrying a *K14CreER* allele [33], which is specifically expressed in the epidermis, to generate *K14Cre^ER^*-*Meis1^fl/fl^* progeny in which Meis1 can be rendered non-functional in the skin upon induction with tamoxifen. One subcutaneous injection of tamoxifen into *K14Cre^ER^*-*Meis1^fl/fl^* mice was sufficient to disrupt the floxed *Meis1* locus in dorsal back skin and tail (**Fig. S1A**). After confirming that the knockout had indeed been induced in our mouse model, we analyzed the dorsal back skin of *K14Cre^ER^*-*Meis1^fl/fl^* mice (n = 5) at one week after tamoxifen induction and compared it with the dorsal back skin of treated *Meis1^fl/fl^* (n = 3) littermates by HE (hematoxylin and eosin) analysis. We observed that *K14Cre^ER^*-*Meis1^fl/fl^* mice had massively thickened epithelium in comparison to their *Meis1^fl/fl^* littermates (**Fig. 2A and 2B**), suggesting that, in the absence of *Meis1, K14Cre^ER^*-*Meis1* ^fl/fl^ mice have increased cell proliferation. Indeed, immunofluorescence analysis with the cell proliferation marker Ki-67 showed an increased number of Ki-67-positive cells in *K14Cre^ER^*-*Meis1* ^fl/fl^ mice, mainly in the basal layer of the epidermis (*P* = 0.0004, *t*-test) (**Fig. 2C, 2D, and 2G**), indicating that *Meis1* deficiency induces hyperproliferation of the epidermis.

To investigate further the requirement for *Meis1* in quiescent epidermal stem cells, we again performed BrdU-chase experiments in *K14Cre^ER^*-*Meis1* ^fl/fl^ mice. BrdU-LRCs were almost undetectable in the bulge of *K14Cre^ER^*-*Meis1* ^fl/fl^ mice (n = 16), compared with the case in the *Meis1* ^fl/fl^ control mice (n = 10) (*P* = 7.939×10^−10,^ *t*-test) **(** **Fig. 2E, 2F, and 2H** **)**. These results demonstrate that the disruption of *Meis1* function results in a reduction in the number of quiescent stem cells and a concomitant increase of differentiation into epidermal cells, as evidenced by the increased thickening of the epidermis of *K14Cre^ER^*-*Meis1^fl/fl^* mice.

### *Meis1* is required for benign tumor development

**Figure 3:**
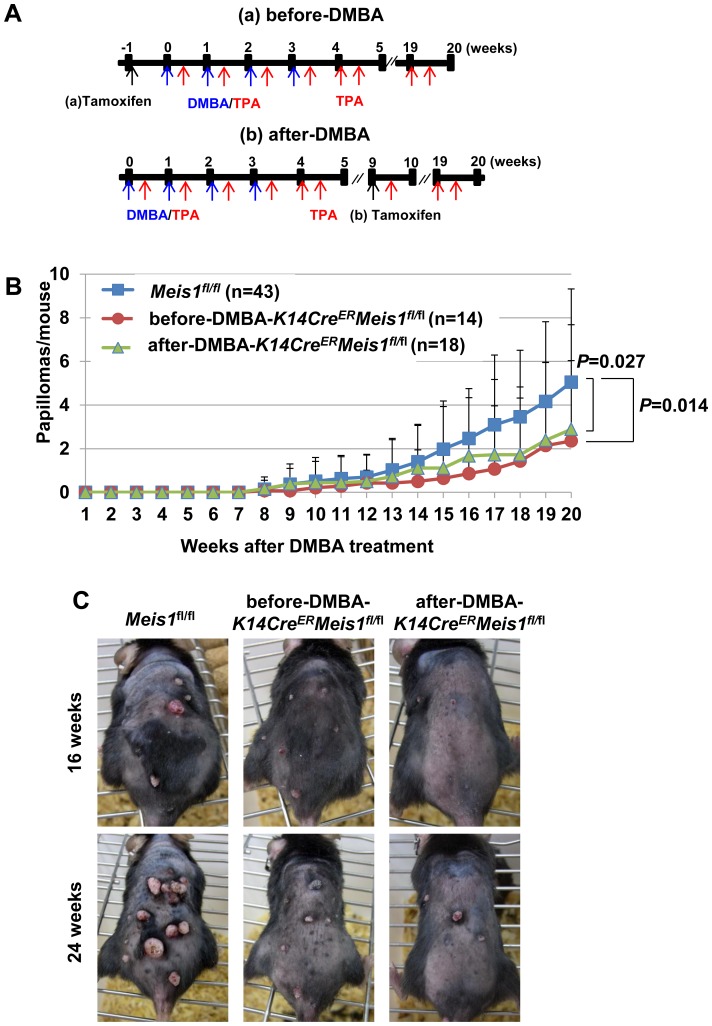
Responsiveness of *K14Cre^ER^-Meis1^fl/fl^* mice to two-stage skin carcinogenesis. (A) Experimental strategy for analysis of the two-stage skin carcinogenesis using DMBA/TPA. “Before-DMBA” means tamoxifen was administered one day before topical DMBA application. “After-DMBA” means tamoxifen was administered nine weeks after topical DMBA application. (B) Comparison of DMBA/TPA-induced papilloma number per mouse among *Meis1^fl/fl^* (n = 43), before-DMBA-*K14Cre^ER^-Meis1^fl/fl^* (n = 14), and after-DMBA-*K14Cre^ER^-Meis1^fl/fl^* (n = 18). Error bars are the standard deviations (S.D.). The *P*-value was calculated for papilloma number at 20 weeks by *t*-test. (C) Representative photographs of mice upon the treatment of DMBA-TPA. *Meis1^fl/fl^* mouse (left), before-DMBA-*K14Cre^ER^-Meis1^fl/fl^* mouse (middle), and after-DMBA-*K14Cre^ER^-Meis1^fl/fl^* mouse (right) at 16 and 24 weeks after initiation.

**Figure 4:**
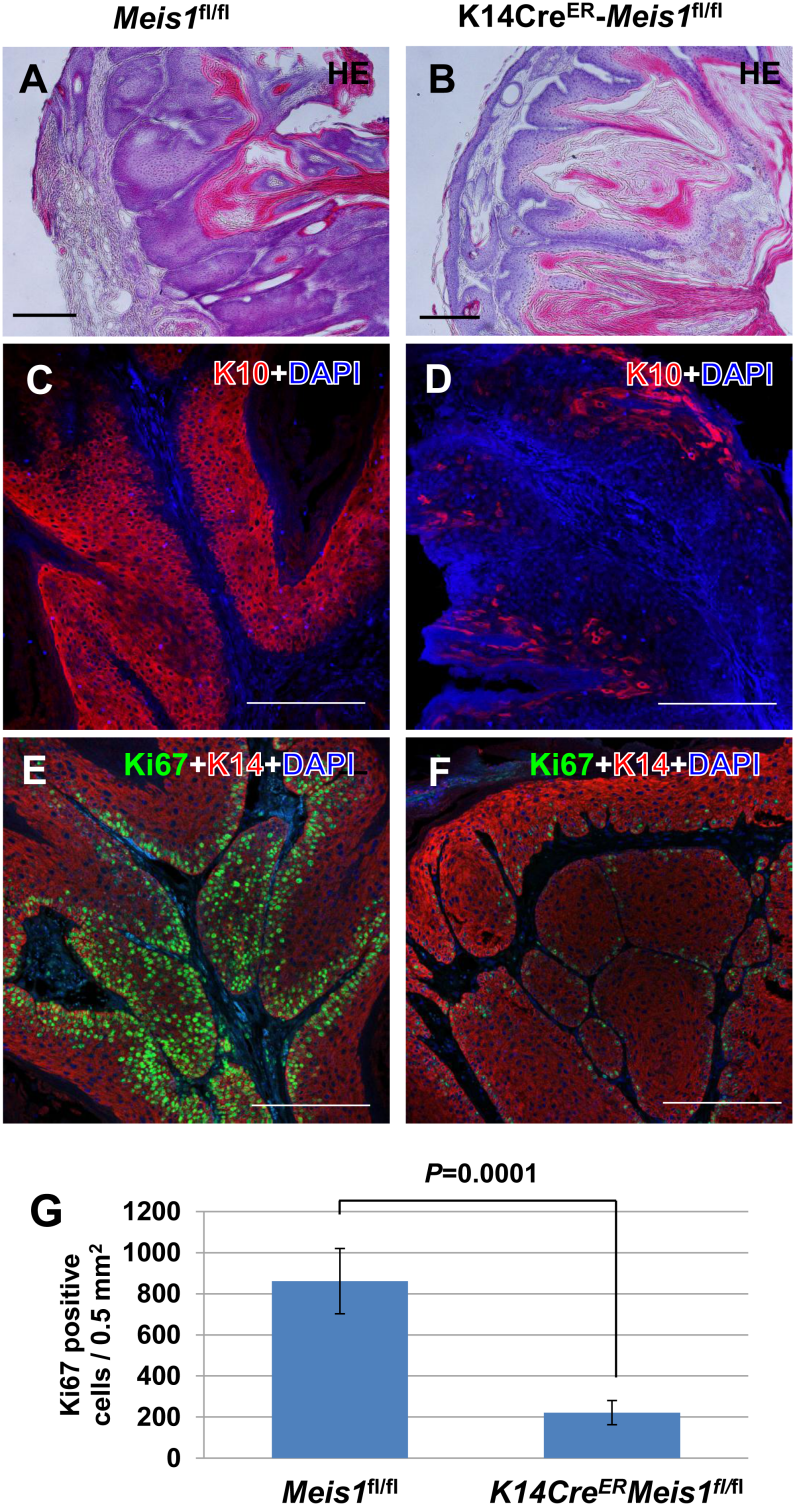
Disruption of *Meis1* leads to limited growth in papillomas. HE staining for papillomas in (A) *Meis1^fl/fl^* mouse and (B) before-DMBA-*K14Cre^ER^-Meis1^fl/fl^* mouse. Representative immunostaining pattern of K10 (red) in papillomas from (C) control *Meis1^fl/fl^* mice and (D) before- *K14Cre^ER^-Meis1^fl/fl^* mice 20 weeks after initiation. Cells were counterstained with DAPI (blue). Scale bars, 100 µm. Representative double immunostaining pattern of Ki-67-positive cells (green) and K14 (red) in papillomas from (E) control *Meis1^fl/fl^* mice and (F) before-*K14Cre^ER^-Meis1^fl/fl^* mice 20 weeks after initiation. Cells were counterstained with DAPI (blue). Scale bars, 100 µm. (G) Number of Ki67-positive cells per 0.5 mm^2^ in papilloma sections of control *Meis1^fl/fl^* and before-*K14Cre^ER^-Meis1^fl/fl^* mice. Error bars are the standard deviations (S.D.). The *P*-value was calculated for the number of Ki67-positive cells by *t*-test.

To investigate the role of the *Meis1* gene in skin carcinogenesis, we subjected 32 *K14Cre^ER^*-*Meis1^fl/fl^* mice and 43 *Meis1^fl/fl^* control mice to the DMBA/TPA chemical carcinogenesis protocol and monitored their tumor development for a period of 35 weeks. In the standard DMBA/TPA protocol, mice are treated once with DMBA, followed by repeated application of TPA. However, since the *K14Cre^ER^*-*Meis1^fl/fl^* mice were in a C57BL6/J background, which is known to be resistant to tumor induction by DMBA/TPA chemical treatment [20], we modified the standard DMBA/TPA protocol and included four additional rounds of DMBA-TPA treatment to overcome the chemical resistance of C57BL6/J mice (**Fig. 3A** **, Methods, Okumura K** ***et al*** **. unpublished data**). The histology of these papillomas was confirmed to be the same as that induced by the standard DMBA/TPA protocol (**Figure 4A, B**). We also checked *Hras* mutation by restriction digestion (**Fig. S1B**). *Hras* is known to be mutated in more than 80 percent of papillomas induced by the standard protocol [20]. Almost the same frequency of *Hras* mutation was detected in papillomas induced by this modified protocol.

Tamoxifen was injected into 14 of the 32 *K14Cre^ER^*-*Meis1^fl/fl^* mice and 22 of the 43 *Meis1^fl/fl^* mice one week before DMBA treatment, hereafter referred to as the “before-DMBA” group, and into 18 of the 32 *K14Cre^ER^*-*Meis1^fl/fl^* mice and 21 of the 43 *Meis1^fl/fl^* mice nine weeks after the first DMBA treatment, hereafter referred to as the “after-DMBA” group (**Fig. 3A**).

Papillomas are visually identified by their cauliflower-like projections that may appear white or normal-colored and may be pedunculated or sessile. Carcinomas are distinguished from papillomas by their flattened appearance. For each mouse, we documented the number of papillomas present at 20 weeks after tumor initiation as well as the number of carcinomas present at 35 weeks after tumor promotion. In addition, the size of each papilloma was measured and recorded. In the control mice, *Meis1^fl/fl^* (before- and after-DMBA combined) mice developed 5.05±4.27 papillomas at 20 weeks after initiation (**Fig. 3B, 3C**). In contrast, before-DMBA-*K14Cre^ER^*-*Meis1^fl/fl^* and after-DMBA-*K14Cre^ER^*-*Meis1^fl/fl^* mice developed 2.36±3.68 and 2.89±4.79 papillomas, respectively (**Fig. 3B, 3C**), indicating that the total number of papillomas at 20 weeks was significantly lower than in control *Meis1^fl/fl^* mice (*Meis1^fl/fl^* vs. before-DMBA, *P* = 0.014; *Meis1^fl/fl^* vs. after-DMBA, *P* = 0.027; all *t*-tests) (**Fig. 3B, 3C**). These results suggest that *Meis1* functions in papilloma development.

### *Meis1* functions to maintain cell proliferation in benign tumors

To investigate the mechanism by which *Meis1* supports papilloma development, we analyzed the papillomas in *K14Cre^ER^*-*Meis1^fl/fl^* and *Meis1^fl/fl^* mice by standard histology methods. HE staining of papillomas from knockout *K14Cre^ER^*-*Meis1^fl/fl^* and control *Meis1^fl/fl^* showed no significant morphological differences (**Fig. 4A, 4B**). Next, we conducted immunostaining with keratin 10 (K10/Krt10), which is a cytoplasmic intermediate filament protein expressed in the suprabasal layer of the normal epidermis. It is also a marker for differentiated tumor cells in papillomas. As a result, we observed almost no K10-positive cells in papillomas of *K14Cre^ER^*-*Meis1^fl/fl^* mice (n = 5) compared with the case in papillomas of the control *Meis1* ^fl/fl^ mice (n = 6) (**Fig. 4C, 4D**). These results suggest that Meis is required for maintaining more differentiated tumor cells in papillomas. We then performed immunostaining with the cell proliferation marker Ki-67 to investigate the cell proliferation capacities of papillomas. We observed fewer Ki-67-positive cells in papillomas of *K14Cre^ER^*-*Meis1^fl/fl^* mice (n = 5) than in papillomas of the control *Meis1* ^fl/fl^ mice (n = 6) (*P* = 0.0001, *t*-test) (**Fig. 4E–4G**). These observations indicate that papillomas from *Meis1*-deficient mice grow more slowly when *Meis1* is present. Taken together, our findings suggest that *Meis1* supports both the development and the growth of papillomas by maintaining cell proliferation.

### *Meis1* is required for malignant conversion

**Figure 5:**
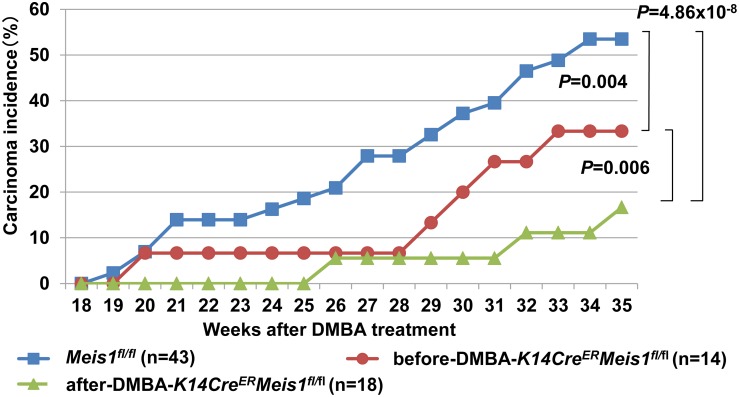
Disruption of *Meis1* inhibits malignant conversion. Comparison of DMBA/TPA-induced carcinoma incidence among *Meis1^fl/fl^* (n = 43) (shown in blue), before-DMBA-*K14Cre^ER^-Meis1^fl/fl^* (n = 14) (shown in red), and after-DMBA-*K14Cre^ER^-Meis1^fl/fl^* (n = 18) (shown in green). The *P*-value was calculated for carcinoma incidence at 35 weeks by Fisher’s test.

To investigate the effect of *Meis1* deletion on carcinoma development, we monitored 32 chemically treated *K14Cre^ER^*-*Meis1^fl/fl^* mice and 43 chemically treated *Meis1^fl/fl^* mice up to 35 weeks after tumor initiation. We observed that *K14Cre^ER^*-*Meis1^fl/fl^* mice have a lower incidence of carcinoma development as well as a later onset of carcinoma development compared with control *Meis1^fl/fl^* mice (**Fig. 5**).

We observed that the *K14Cre^ER^*-*Meis1^fl/fl^* (after-DMBA) mice developed the first carcinoma at 26 weeks after initiation, while control *Meis1^fl/fl^* mice developed the first carcinoma at 19 weeks. In addition, we observed that less than 20 percent of *K14Cre^ER^*-*Meis1^fl/fl^* (after-DMBA) mice had developed carcinoma at 35 weeks, whereas 53 percent of control *Meis1* ^fl/fl^ mice had done so. This difference between *K14Cre^ER^*-*Meis1^fl/fl^* (after-DMBA) and control *Meis1* ^fl/fl^ mice was statistically significant (*P* = 4.86×10^−8^, Fisher’s test) (**Fig. 5**).

In the *K14Cre^ER^*-*Meis1^fl/fl^* (before-DMBA), we observed that the first carcinoma developed at 20 weeks, while control *Meis1^fl/fl^* mice developed the first carcinoma at 19 weeks. Furthermore, 33 percent of *K14Cre^ER^*-*Meis1* ^fl/fl^ (before-DMBA) mice had developed carcinoma at 35 weeks, whereas 53 percent of control *Meis1* ^fl/fl^ mice had done so. Interestingly, we found that the difference in the number of carcinomas between *K14Cre^ER^*-*Meis1^fl/fl^* (after-DMBA) and *K14Cre^ER^*-*Meis1^fl/fl^* (before-DMBA) was statistically significant (*P* = 0.006, Fisher’s test), as was the difference between *K14Cre^ER^*-*Meis1^fl/fl^* (before-DMBA) and control *Meis1* ^fl/fl^ mice (*P* = 0.004, Fisher’s test) (**Fig. 5**). Our investigations of *Meis1* in tumorigenesis indicate that, in addition to the role in papilloma development, *Meis1* also functions to support the malignant conversion of benign papillomas into malignant tumors.

### *Meis1* expression successively increases with skin tumor progression

**Figure 6:**
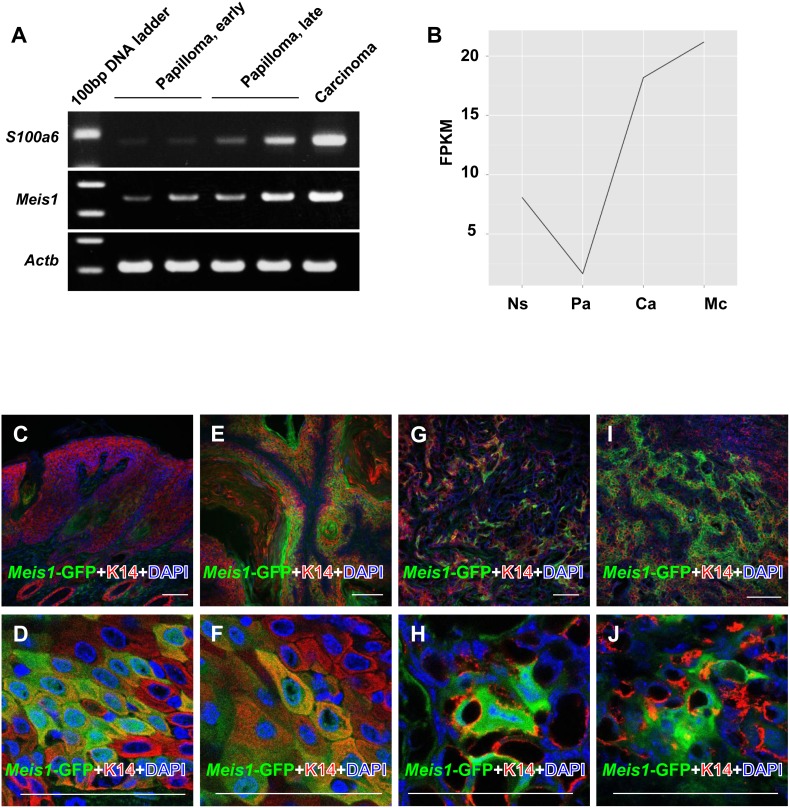
Enhancement of *Meis1* expression in malignant tumors. (A) *Meis1* mRNA expression in skin tumors of *Meis1*-EGFP reporter mice by reverse-transcription PCR. *S100a6* expression is shown as a malignancy marker. *β-Actin* expression is shown as an internal control. “Papilloma, early” means papillomas at 10 weeks after initiation. “Papilloma, late” means papillomas at 18 weeks after initiation. (B) *Meis1* mRNA expression in skin tumors of FVB mice by RNA-seq using next-generation sequencing. See material and methods for more details of FPKM (fragments per kilobase of exon per million mapped fragments) analysis. “Ns” means “normal skin”, “Pa” means “papillomas at 10 weeks after initiation”, “Ca” means “carcinomas in situ”, and “Mc” means “metastatic carcinomas”. (C–N) Immunofluorescence microscopic analysis of papillomas, carcinomas in situ, and metastatic carcinomas induced by DMBA/TPA in *Meis1*-EGFP reporter mice. (C–J) The sections were stained with anti-GFP antibody (green), in combination with anti-K14 antibody (red). Cells were counterstained with DAPI (blue). (C) An early-stage papilloma at 11 weeks after initiation. (D) A magnified image of (C). (E) A late-stage papilloma at 18 weeks after initiation. (F) A magnified image of (E). (G) A carcinoma in situ at 40 weeks after initiation. (H) A magnified image of (G). (I) A metastatic carcinoma at 40 weeks after initiation. (J) A magnified image of (I). Scale bars, 100 µm (C, E, G, I). Scale bars, 50 µm (D, F, H, J).

To assess further the role of *Meis1* in skin carcinogenesis, we examined the expression of *Meis1* in chemically induced papillomas, carcinomas *in situ,* and metastatic carcinomas from wild-type FVB/N mice by semi-quantitative RT-PCR and RNA sequencing (Aoto *et al*., *unpublished data*). The semi-quantitative RT-PCR study showed that *Meis1* mRNA expression level increased during skin tumor development and progression (**Fig. 6A**). The expression of *S100a6*, one of the S100 family members, was checked in parallel as a malignancy marker [34], (**Fig. 6A**). For the RNA sequencing assay, the fragment number of cDNA containing the *Meis1* gene was counted and plotted as FPKM (fragments per kilobase of exon per million mapped fragments, **Fig. 6B**). Data from RNA sequencing were similar to those detected by RT-PCR analysis (**Fig. 6A, 6B**). Specifically, both RNA sequencing and RT-PCR analysis indicated that *Meis1* expression is successively increased from papillomas to carcinomas *in situ* to metastatic carcinomas. Collectively, these results indicate a proto-oncogenic role for *Meis1.*

We next investigated Meis1 expression in chemically induced papillomas, carcinomas *in situ*, and metastatic carcinomas of *Meis1*-EGFP mice by immunofluorescence (**Fig. 6C–6J**). Tumors were harvested at 11, 18, and 35 weeks after initiation. We co-stained the tumors for *Meis1*-EGFP and keratin 14 (K14/Krt14) expression. K14 is a cytoplasmic intermediate filament protein expressed widely in the normal epidermis and skin tumors. We observed co-expression of *Meis1*-EGFP and K14 in epidermal cell layers of early papillomas at 11 weeks after initiation (**Fig. 6C and 6D**). In the papillomas harvested at 18 weeks, we observed that the range of *Meis1*-EGFP expression expanded beyond the basal layer cells of the epidermis (**Fig. 6E and 6F**). In carcinomas *in situ*, we found that *Meis1*-EGFP was co-expressed with K14 (**Fig. 6G and 6H**). In metastatic carcinomas, *Meis1*-EGFP was expressed throughout the metastatic carcinoma (**Fig. 6I and 6J**). Our observations indicate that *Meis1*-EGFP expression successively increases from the basal cell layer to the entire tumor as skin tumor development progresses.

Epithelial-to-mesenchymal transition (EMT) is a biological process that occurs during tumor development that transforms the existing epithelial tissue of cancer cells into mesenchymal tissue as a benign tumor, such as a papilloma developing into a malignant tumor such as a carcinoma. To determine whether or not the observed increase of *Meis1* expression is associated with the development of increased mesenchymal tissue owing to EMT, we analyzed carcinomas in which the expansion of mesenchymal tissue is known to occur. Immunofluorescence analysis was performed to determine whether *Meis1*-EGFP is co-expressed with epithelial or mesenchymal tissue markers. In both carcinomas *in situ* and metastatic carcinomas, we observed that *Meis1*-EGFP was co-expressed with the epithelial basal marker K14, but not with the mesenchymal marker vimentin (**Fig. S3A–S3D**). Taken together, these results indicate that the successive increase of *Meis1* expression during skin tumor development is not due to the increase in mesenchymal tissue associated with EMT, but rather indicates oncogenic potential of Meis1 during tumorigenesis.

### *Meis1* is localized to a non-stem cell region in benign tumors

**Figure 7:**
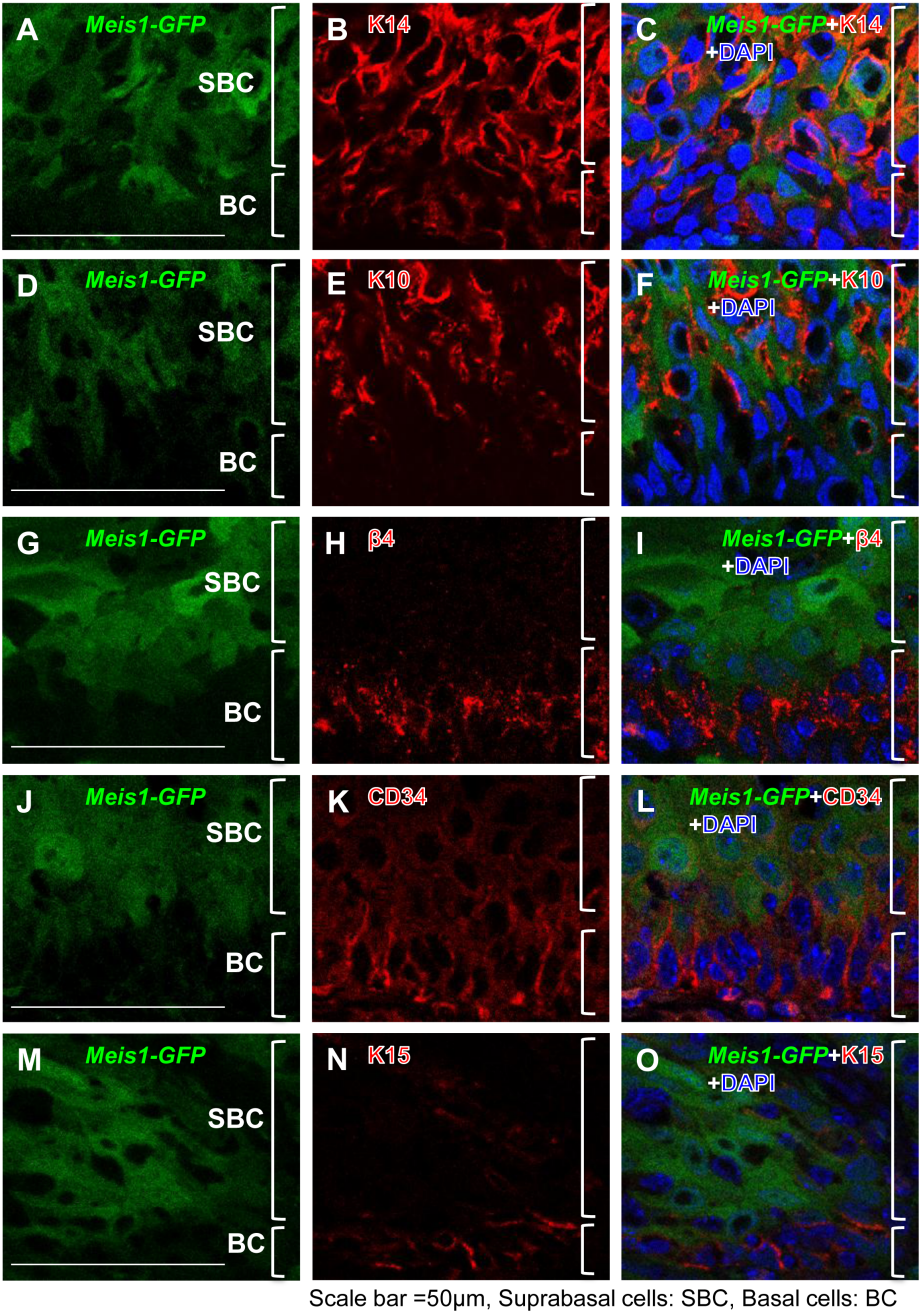
Meis1 protein localization in papillomas. (A–O) Immunofluorescence microscopic analysis of papillomas induced by DMBA/TPA in *Meis1*-EGFP reporter mice. The papilloma sections at 18 weeks after initiation were stained with the anti-GFP antibody (green), in combination with anti-K14, K10, β4-integrin, CD34, and K15 antibodies (red). Cells were counterstained with DAPI. (A) *Meis1*-EGFP (green) fluorescence and (B) K14 (red) are shown. (C) The merged image of *Meis1*-EGFP with K14. (D) *Meis1*-EGFP (green) fluorescence and (E) K10 (red) are shown. (F) The merged image of *Meis1*-EGFP with K10. (G) *Meis1*-EGFP (green) fluorescence and (H) β4-integrin (red) are shown. (I) The merged image of *Meis1*-EGFP with β4-integrin. (J) *Meis1*-EGFP (green) fluorescence and (K) CD34 (red) are shown. (L) The merged image of *Meis1*-EGFP with CD34. (M) *Meis1*-EGFP (green) fluorescence and (N) K15 (red) are shown. (O) The merged image of *Meis1*-EGFP with K15. Abbreviations: “SBC” means suprabasal cells (A, D, G, J, and M) and “BC” means basal cells (A, D, G, J, and M). Scale bars, 50 µm (A, D, G, J, and M).

To investigate further the role of *Meis1* in cancer tissues, we analyzed Meis1 expression in chemically induced papillomas from *Meis1*-EGFP reporter mice by immunostaining. We co-stained for *Meis1*-EGFP with K14, K10, and cancer stem cell markers such as β4 integrin, CD34, and K15 (**Fig. 7A–7O**). In contrast to Meis1 expression in normal epidermis (**Fig. 1**), we found that *Meis1*-EGFP was not co-expressed with β4 integrin, CD34, or K15 in the tumor basal layers, where epidermal cancer stem cells are thought to reside in papillomas (**Fig. 7G–7O**). Interestingly, in papillomas, we observed that instead of localizing to a stem cell compartment, *Meis1*-EGFP was co-expressed with K10 (**Fig. 7D–7F**), which is a marker for differentiated tumor cells in papillomas. The change in Meis1 localization in malignant tissues suggests that a functional switch in *Meis1* roles occurs when the epidermis is transformed from normal into hyperproliferative tissue.

## Discussion

**Figure 8:**
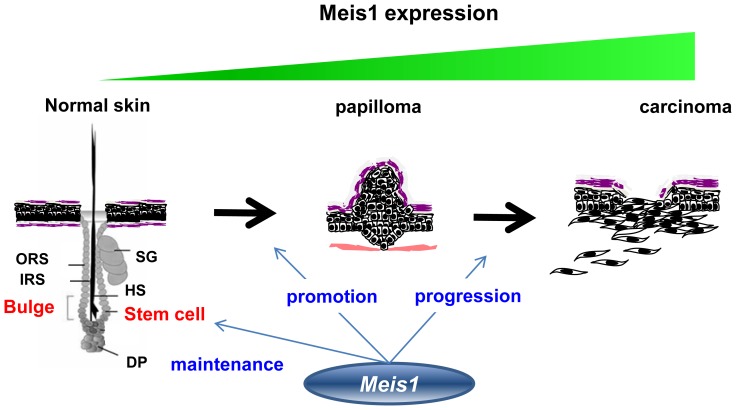
A schematic drawing of *Meis1’s* multiple functions in the process of skin carcinogenesis. We illustrate *Meis1’s* multiple functions in a schematic drawing. In normal skin, *Meis1* has an important role for stem cell maintenance. In papilloma, *Meis1* regulates papilloma growth as well as malignant conversion from papilloma into carcinoma. Abbreviations: “ORS” means “outer root sheath”, “IRS” means “inner root sheath”, “HS” means “hair shaft”, “SG” means “sebaceous gland”, and “DP” means “dermal papilla”.

*Meis1’s* functions and mechanisms in the epithelium remain poorly understood. Here, we present knockout and cell marker studies revealing *Meis1’s* roles in both normal and tumor development. Our investigations of normal tissue indicate that *Meis1* functions to maintain epidermal adult stem cells in the bulge region of the epidermis to maintain homeostasis of the epidermis. In contrast, our studies in the two-stage skin carcinogenesis mouse model indicate that *Meis1* function changes to a more pro-tumorigenic role, supporting tumor development and malignant conversion. Collectively, our findings suggest a multi-function model for *Meis1* in the epidermis (**Fig. 8**).

Here, we show that *Meis1* expression in normal skin is predominantly associated with stem cells of the hair follicle bulge (**Fig. 1**). The hair follicle bulge is one of the stem cell niches of the epidermis [35]. However, the expression of Meis1 is not restricted to the bulge (**Fig. 1**). It is also expressed mainly in temporary portions passing through hair cycles within the hair follicles. Several lines of evidence have suggested the existence of non-LRC-type stem cells, such as Lgr (Leucine-rich repeat-containing G-protein-coupled Receptor)-positive stem cells in the same region within the hair follicles [36]. Further marker staining, such as of Lgr proteins, and mouse cross experiments with LGR-Cre mice will be required for future study. Meis1 is also expressed in the hair bulbs and dermal papillae (**Fig. 1**). It could be involved not only in stem cell maintenance but also in hair growth or maintenance. Nevertheless, our data suggest that disruption of *Meis1* in the epidermis causes reduction of epidermal stem cells in the bulge and then thickened epithelium (**Fig. 2**). This is consistent with the observation that *Meis1* expression is predominantly associated with the stem cells of the hair follicle bulge. Our findings of *Meis1* function in the epidermis are similar to *Meis1* studies in the bone marrow that showed that hematopoietic stem cell (HSC) compartments were severely affected and potentials for colony formation as well as repopulation of lethally irradiated recipient mice were profoundly impaired in *Meis1*-deficient cells, suggesting a critical role of *Meis1* in HSC maintenance [37]. On the basis of these findings, we propose a model in which *Meis1* is essential for maintaining quiescent epidermal stem cells in the hair follicle bulge of the epidermis (**Fig. 8**).

Additionally, we present RT-PCR and RNA sequencing data that show that *Meis1* expression is successively enhanced with skin tumor progression (**Fig. 6**). The observed successive increase in expression of *Meis1* during carcinogenesis, independent of EMT, suggests an oncogenic role for *Meis1* in the two-stage skin carcinogenesis mouse model. Consistent with this conclusion, elevated *Meis1* expression has also been observed in several tumor types including acute myeloid leukemia, lung adenocarcinoma tumors, neuroblastomas, ovarian carcinomas, and nephroblastomas. We propose a model in which *Meis1* has two distinct functions influencing the promotion and progression of skin carcinogenesis (**Figure 8**).

The balance between self-renewal and differentiation of adult stem and progenitor cells maintains tissue homeostasis. Loss of *Meis1* causes reduction of epidermal stem cells in the bulge, hyperproliferative epidermal cells, and then thickened epidermis (**Fig. 2**), indicating that Meis1 drives self-renewal in order to maintain quiescence of epidermal stem cells. However, in the absence of Meis1, the balancing mechanisms are disrupted, and the balance is shifted to differentiation. There are a lot of reports of knockout mice showing abnormal epidermal thickness. In the case of mice with the knockout of genes driving the self-renewal of stem cells, like Meis1, they tend to show thicker epidermis [38], [39], [40]. In particular, aPKCλ (atypical PKCλ) knockout mice show loss of quiescent hair follicle bulge stem cells, a temporary increase in proliferating epidermal cells, and thicker epidermis, which were all seen in our Meis1 study. These two genes might thus have very closely related functions. On the other hand, when the gene expression is restricted to progenitor cells in the epidermis, like for *DNMT1* [41], disruption of the gene does not necessarily result in thicker epidermis. This is probably because of the difference between quiescent stem cells and more rapidly cycling progenitor cells. Because stem cells have higher differentiation potential than progenitor cells, deficiency of a balancing factor driving self-renewal in stem cells causes drastic differentiation into epidermal cells. Furthermore, our investigations of the role of *Meis1* in tumorigenesis revealed an unexpected switch of Meis1 localization from a stem cell region in normal epidermal tissues to a more differentiated region in tumor tissues (**Fig. 7**). Interestingly, a few recent studies have shown that carcinoma stem cell-like cells were capable of forming well-differentiated tumor tissue. Previous studies indicated that epidermal cells of all types including cancer cells were derived from keratinocytes of the bulge region [35], [42]. Another recent study showed that CD34-expressing (a carcinoma stem cell (CSC) marker) cells, residing in DMBA/TPA-induced skin tumors, formed secondary tumors upon transplantation into immunodeficient mice [43]. However, another study showed that well-organized, differentiated papillomas containing CD34, β4-integrin, and K15-expressing cells (CSC markers) gave rise to layers of differentiated tissues [44]. Although tumor-forming potential appears to be maintained in carcinoma stem cells of skin tumors, such an explanation for the role of *Meis1* in tumorigenesis is inconsistent with our findings. The cell marker expression studies in papillomas from *Meis1* knockout mice indicate that *Meis1* is not essential for maintaining CSCs in papillomas, as Meis1 expression in papillomas did not overlap with CSC-like markers (**Fig. 7**). While *Meis1* function is required for maintaining epidermal stem cells in normal skin, our results suggest that *Meis1* loses its stem cell maintenance function and switches to a mechanistically distinct role in tumorigenesis.

It has been known for many years that, in mouse skin carcinogenesis, initiated cells persist throughout the lifetime of the animal, suggesting that the incipient tumor cell is long-lived, a quality of stem cells [45]. LRCs of hair follicles are slow-cycling and long-lived, thus presenting characteristics of stem cells. In carcinogen-treated mouse skin, carcinogen-DNA adducts persist in these cells [46], and even after ablation of cycling cells in the epidermis with a chemotherapeutic drug prior to DMBA treatment, the rate of carcinoma formation is unchanged, indicating that tumor initiation occurs in quiescent stem cells rather than rapidly proliferating (TA) cells [47]. *Meis1* was predominantly expressed in quiescent epidermal stem cells and essential for maintaining these cells. In addition, when the knockout of Meis1 was induced before DMBA tumor initiation, the number of papillomas significantly decreased. These results clearly suggest that tumor initiation occurs in quiescent stem cells and Meis1 is indispensable for maintaining these tumor-initiating (quiescent stem) cells. To investigate further *Meis1* functions in skin carcinogenesis, we subjected tamoxifen-inducible, conditional knockout *K14Cre^ER^-Meis1^fl/fl^* mice to DMBA/TPA chemical carcinogenesis using two different treatment schemes. One set of mice were treated with tamoxifen, such that the knockout of *Meis1* was induced one week before DMBA tumor initiation (referred to as “before-DMBA”) and the other set of mice were treated with tamoxifen, such that the knockout of *Meis1* was induced nine weeks after DMBA tumor initiation (referred to as “after-DMBA”) (see **Fig. 3A**). Both the “before-DMBA” and the “after-DMBA” *K14Cre^ER^-Meis1^fl/fl^* treated mice showed significant decreases in the number of papillomas compared with control *Meis1^fl/fl^* mice (**Fig. 3B**). However, the mechanisms driving the observed papilloma reduction in the “before-DMBA” mice are likely to differ from those in the “after-DMBA” mice. Given the absence of epidermal stem cells in *K14Cre^ER^-Meis1^fl/fl^* mice (**Fig. 2E, 2F** **)**, taken together, this suggests that the decrease in papilloma in the “before-DMBA” mice is probably due to the decrease of epidermal stem cells. Meanwhile, the observed increase in *Meis1* expression in early and late papillomas (**Fig. 6A, 6B**) suggests that the decrease in papilloma in the “after-DMBA” mice was due to *Meis1*’s function in papilloma maintenance.

When we examined the effects on malignant conversion in the “before-DMBA” and “after-DMBA” *K14Cre^ER^-Meis1^fl/fl^* mice, we observed that both of these groups showed significant decreases in the number of SCCs compared with control *Meis1* ^fl/fl^ mice (**Fig. 5**). In addition, we found that the “after-DMBA” mice showed significantly lower incidence of SCC than the “before-DMBA” mice at 35 weeks (**Fig. 5**). Collectively, our results show that *Meis1* has an oncogenic role in the epidermis, where it supports papilloma development and malignant conversion of skin tumors. Furthermore, the results from our “before-DMBA” and “after-DMBA” treatment studies provide further insight on the fundamental question of when malignant conversion is determined in the DMBA/TPA carcinogenesis mouse model. Our results suggest that malignant conversion is determined in this model no later than nine weeks after tumor initiation.

Our results also suggest that targeting *Meis1* in skin tumors could be a powerful strategy for the treatment of skin cancer as well as other epithelial tumors in which *Meis1* appears to have an oncogenic role, such as endometrial and ovarian cancers.

## Materials and Methods

### Mice

This study was carried out in strict accordance with the recommendations in the Guide for the Care and Use of Laboratory Animals of the Ministry of Education, Culture, Sports, Science, and Technology of Japan. The protocol was approved by the Committee on the Ethics of Animal Experiments of Chiba Cancer Center (Permit Number: 13–18). All efforts were made to minimize suffering. Details on the generation of mice carrying the floxed allele of the *Meis1* gene (*Meis1^fl/fl^*) are described elsewhere [11], [37]. *Meis1* ^fl/fl^ mice were mated to *K14-Cre^ER^* transgenic mice [48]. *K14-Cre^ER^* transgenic mice were obtained from The Jackson Laboratory (JaX 005107). *Meis1-*EGFP BAC-transgenic reporter mice, generated by the GENSAT BAC transgenic project [32], were obtained from the Mutant Mouse Regional Resource Center. All mice had a C57BL/6J background. Mice carrying alleles or transgenes were maintained and genotyped as previously described. To disrupt *Meis1*, 8-week-old *K14Cre^ER^*-*Meis1^fl/fl^* and *Meis1^fl/fl^* mice were injected subcutaneously 1 time with 0.5 mg of tamoxifen (final concentration 5 mg/ml, Sigma-Aldrich) that had been prepared following the manufacturer’s instructions by completely dissolving tamoxifen into 250 µl of 100% ethanol at 55°C, adding 4750 µl of sunflower oil, and mixing them well by vortexing.

### Skin carcinogenesis

7,12-Dimethylbenz(a)anthracene (DMBA) was purchased from Sigma Japan, and 12-*O*-tetradecanoylphorbol-13-acetate (TPA) was purchased from Calbiochem. DMBA is used as a carcinogen and TPA as a promoter. A total of 43 *Mes1* ^fl/fl^ mice and 32 *K14Cre^ER^*-*Meis1^fl/fl^* mice were treated according to the modified two-stage carcinogenesis protocol. At 8–10 weeks of age, the backs of the mice were carefully shaved with an electric clipper. Two days after shaving, DMBA (25 µg per mouse in 200 µl of acetone) was applied to shaved dorsal back skin. Three days after the first DMBA treatment, TPA (10 µg per mouse in 200 µl of acetone) was applied. After four rounds of this single DMBA and TPA treatment, the mice were treated with TPA twice weekly for 20 weeks. Papilloma number and size (mm in diameter) of each papilloma were recorded from 10 weeks up to 20 weeks, and carcinoma development was monitored up to 35 weeks post-TPA treatment.

### Immunofluorescence

The dorsal back skin or tumors were fixed in 4% paraformaldehyde at 4°C overnight. Frozen skin and tumor embedded in OCT compound (Sakura Finetek) were cut into 10 µm sections. In contrast, dehydrated samples were embedded in paraffin and sectioned as 10 µm slices, which were stained with hematoxylin and eosin. The endogenous peroxidase activity in the specimens was blocked by treatment with 0.3% H_2_O_2_ and samples were then rinsed with PBS. Sections were incubated with primary antibodies diluted in blocking buffer overnight at 4°C. The following primary antibodies were used: rabbit anti-EGFP (1∶100, Molecular Probes, Invitrogen), goat anti-EGFP (1∶50, Santa Cruz), rat anti-CD34 (1∶200, eBioscience), rabbit anti-keratin 14 (1∶500, Covance Research), rabbit anti-K10 (1∶100, Covance Research), rabbit anti-keratin 15 (1∶100, Abcam), rat anti-Ki-67 (1∶200, DakoCytomation), rat anti-CD104 (1∶100, BioLegend), and rabbit anti-vimentin (1∶100, Abcam). Secondary antibodies were Alexa Fluor 488-conjugated anti-rat antibody (1∶100, Molecular Probes, Invitrogen) and Alexa Fluor 568-conjugated anti-rabbit antibody (1∶100, Molecular Probes, Invitrogen). Nuclei were counterstained with Hard Set Mounting Medium with DAPI (Vector). All fluorescence images were obtained with a Leica TCS SPE confocal microscope equipped with a DMI4000B (10X/0.40, 20X/0.70, and 40x/1.25 oil immersion objective).

### Classification between papillomas and carcinomas

The majority of papillomas were determined by visual inspection. Papillomas appeared as outgrowths on the dorsal skin of mice. Some of the papillomas began to convert to carcinomas by becoming flatter on the skin and penetrating deeper into the dermis. They were normally easily distinguishable from one another. However, in some intermediate-type tumors, we prepared a paraffin section and confirmed the histology.

### BrdU chase experiments

For chase experiments, BrdU (Sigma-Aldrich) was administered by peritoneal injection of postnatal day 24 (P24) mice (40 µg per gram of body weight) on three consecutive days. Mice were then chased for five weeks. Meanwhile, P44 mice were treated with tamoxifen. After the five weeks of chase, skin tissues were fixed with 4% paraformaldehyde and stained with rat anti-BrdU (Abcam) antibody.

### RT-PCR

Total RNA was isolated from normal skin, papillomas, carcinomas, and metastatic carcinomas of mice after 40 weeks of DMBA/TPA treatment using TRIzol (Invitrogen) following the manufacturer’s protocol. cDNA was generated with the iScript Select cDNA Synthesis Kit (Bio-Rad) using 0.1 µg of DNase-pretreated total RNA. The cDNA was amplified for 40 cycles (94°C for 30 sec, 50°C for 30 sec, 72°C for 30 sec) using Prime Taq DNA Polymerase Kit (Genet Bio). Primer sets used for amplification of the *Meis1* gene were 5′-GCA AAG TAT GCC AGG GGA GTA-3′ and 5′-TCC TGT GTT AAG AAC CGA GGG-3′. Primer sets used for amplification of the *S100a6* gene were 5′-CAG TGA TCA GTC ATG GCA TGC CCT C-3′ and 5′-CAT TTT ATT TCA GAG CTT CAT TGT AG-3′. The products were subjected to agarose gel electrophoresis. cDNA integrity was confirmed using *β-actin*.

### Gene expression analysis by RNA-Seq

Gene expression levels were estimated using FPKM (expected fragments per kilobase of transcript per million fragments sequenced) with Cufflinks [49]. RNA-Seq reads were aligned to the mouse genome sequence (GRCm38-release71) with a gene annotation file using TopHat2 [50], and the FPKM value for each gene was estimated based on the mapping results obtained using Cufflinks.

### *Hras* mutation analysis

DNA was prepared from papillomas of *Meis1^fl/fl^* and *K14Cre^ER^-Meis1^fl/fl^* mice. Exon 2 of the *Hras* gene was amplified as previously described [51]. Amplified 207-bp fragments were digested with a restriction enzyme, *XbaI*, at 37°C for 4 h and electrophoresed in a 4% Nusieve 3∶1 agarose gel.

### Statistical analysis

Statistical significance was calculated by the unpaired two-tailed Student’s *t*-test or 2×2 Chi square test (Fisher’s test). A *P*-value<0.05 was considered statistically significant and a *P*-value<0.01 was considered highly statistically significant.

## Supporting Information

Figure S1 ***Meis1*** **deletion and** ***Hras*** **mutation analysis.** (A) Confirmation of *Meis1* deletion in *K14Cre^ER^*-*Meis1^fl/fl^* mice. Skin DNA prepared from *K14Cre^ER^*-*Meis1^fl/fl^* mice that were either treated (+) or untreated (−) with TAM (tamoxifen) was subjected to PCR analysis using primer pairs described elsewhere [37]. DNA from *Meis1^fl/fl^* mice was used as a control. FA denotes floxed allele. KOA denotes knockout allele. (B) *XbaI* digests of PCR products in the *Hras* gene. *XbaI* was used to detect the point mutation at codon 61 of the *Hras* gene induced by DMBA. After amplification of *Hras* exon 2 as previously described [51], PCR products from the mutant *Hras* allele produce 116- and 91-bp fragments after *XbaI* single digestion.
(TIF)

Figure S2 **Disruption of Meis1 decreased BrdU-LRCs in the bulge.** (A, B) Representative double immunostaining pattern of BrdU-LRC (green) and K14 (red) in the skin from control *Meis1^fl/fl^* mice (A) and *K14Cre^ER^-Meis1^fl/fl^* mice (B) one week after TAM treatment. Cells were counterstained with DAPI (blue). For chase experiments, BrdU was administered by peritoneal injection. See material and methods for more details of the BrdU chase experiments. (C–F) Immunofluorescence analysis to localize *Meis1*-EGFP-positive cells in adult mouse skin. Dorsal back skin sections from 8-week-old *Meis1*-EGFP reporter mice were stained with anti-GFP antibody, in combination with anti-BrdU antibody. Cells were counterstained with DAPI. (C–E) DAPI (blue), *Meis1*-EGFP (green) fluorescence, and BrdU-LRC (red) are shown. (F) The merged image of *Meis1*-EGFP with BrdU-LRC. Abbreviations: “Bu” means “bulge” and “Dp” means “dermal papilla”. Scale bars, 100 µm.
(TIF)

Figure S3 **Expression of Meis1 did not overlap with vimentin in malignant tumors.** Immunofluorescence microscopic analysis of papillomas, carcinomas in situ, and metastatic carcinomas induced by DMBA/TPA in *Meis1*-EGFP reporter mice. (A–D) The sections were stained with anti-GFP antibody (green), in combination with anti-vimentin antibody (red). Cells were counterstained with DAPI. (A) A carcinoma in situ at 40 weeks after initiation. (B) A magnified image of (A). (C) A metastatic carcinoma at 40 weeks after initiation. (D) A magnified image of (C). Scale bars, 100 µm (A, C). Scale bars, 50 µm (B, D).
(TIF)

## References

[1] Moskow JJ, Bullrich F, Huebner K, Daar IO, Buchberg AM (1995) Meis1, a PBX1-related homeobox gene involved in myeloid leukemia in BXH-2 mice. Mol. Cell. Biol. 15: 5434–5443. 10.1128/mcb.15.10.5434 PMC230793 7565694

[2] Rozovskaia T, Feinstein E, Mor O, Foa R, Blechman J, et al (2001) Upregulation of Meis1 and HoxA9 in acute lymphocytic leukemias with the t(4: 11) abnormality. Oncogene. 20: 874–878. 10.1038/sj.onc.1204174 11314021

[3] Azcoitia V, Aracil M, Martínez-A C, Torres M (2005) The homeodomain protein Meis1 is essential for definitive hematopoiesis and vascular patterning in the mouse embryo. Dev. Biol. 280: 307–320. 10.1016/j.ydbio.2005.01.004 15882575

[4] Hisa T, Spence SE, Rachel RA, Fujita M, Nakamura T, et al (2004) Hematopoietic, angiogenic and eye defects in Meis1 mutant animals. EMBO. J. 23: 450–459. 10.1038/sj.emboj.7600038 PMC1271748 14713950

[5] Mann RS, Affolter M (1998) Hox proteins meet more partners. Curr. Opin. Genet. Dev. 8: 423–429. 10.1016/s0959-437x(98)80113-5 9729718

[6] Maeda R, Mood K, Jones TL, Aruga J, Buchberg AM, et al (2001) Xmeis1, a protooncogene involved in specifying neural crest cell fate in Xenopus embryos. Oncogene. 20: 1329–1342. 10.1038/sj.onc.1204250 11313877

[7] Capdevila J, Tsukui T, Rodríquez Esteban C, Zappavigna V, et al (1999) Control of vertebrate limb outgrowth by the proximal factor Meis2 and distal antagonism of BMPs by Gremlin. Mol. Cell. 4: 839–849. 10.1016/s1097-2765(00)80393-7 10619030

[8] Heine P, Dohle E, Bumsted-O’Brien K, Engelkamp D, Schulte D (2008) Evidence for an evolutionary conserved role of homothorax/Meis1/2 during vertebrate retina development. Development. 135: 805–811. 10.1242/dev.012088 18216174

[9] Bessa J, Tavares MJ, Santos J, Kikuta H, Laplante M, et al (2008) Meis1 regulates cyclin D1 and c-myc expression, and controls the proliferation of the multipotent cells in the early developing zebrafish eye. Development. 135: 799–803. 10.1242/dev.011932 18216175

[10] Tucker ES, Lehtinen MK, Maynard T, Zirlinger M, Dulac C, et al (2010) Proliferative and transcriptional identity of distinct classes of neural precursors in the mammalian olfactory epithelium. Development. 137: 2471–2481. 10.1242/dev.049718 PMC2927697 20573694

[11] Hirayama T, Asano Y, Iida H, Watanabe T, Nakamura T, et al (2014) *Meis1* is required for the maintenance of postnatal thymic epithelial cells. Plos one. 9: e89885. 10.1371/journal.pone.0089885 PMC3942356 24594519

[12] Fernandez P, Carretero J, Medina PP, Jimenez AI, Rodriguez-Perales S, et al (2004) Distinctive gene expression of human lung adenocarcinomas carrying LKB1 mutations. Oncogene. 23: 5084–5091. 10.1038/sj.onc.1207665 15077168

[13] Geerts D, Revet I, Jorritsma G, Schilderink N, Versteeg R (2005) MEIS homeobox genes in neuroblastoma. Cancer. Lett. 228: 43–50. 10.1016/j.canlet.2005.01.047 15919149

[14] Jones TA, Flomen RH, Senger G, Nizetić D, Sheer D (2000) The homeobox gene MEIS1 is amplified in IMR-32 and highly expressed in other neuroblastoma cell lines. Eur. J. Cancer. 36: 2368–2374. 10.1016/s0959-8049(00)00332-4 11094311

[15] Spieker N, van Sluis P, Beitsma M, Boon K, van Schaik BD, et al (2001) The MEIS1 oncogene is highly expressed in neuroblastoma and amplified in cell line IMR32. Genomics 71: 214–221. 11161815 10.1006/geno.2000.6408

[16] Crijns AP, de Graeff P, Geerts D, Ten Hoor KA, Hollema H, et al (2007) MEIS and PBX homeobox proteins in ovarian cancer. Eur. J. Cancer. 43: 2495–2505. 10.1016/j.ejca.2007.08.025 17949970

[17] Dekel B, Metsuyanim S, Schmidt-Ott KM, Fridman E, Jacob-Hirsch J, et al (2006) Multiple imprinted and stemness genes provide a link between normal and tumor progenitor cells of the developing human kidney. Cancer. Res. 66: 6040–6049. 10.1158/0008-5472.CAN-05-4528 16778176

[18] Chen JL, Li J, Kiriluk KJ, Rosen AM, Paner GP, et al (2012) Deregulation of a Hox protein regulatory network spanning prostate cancer initiation and progression. Clin. Cancer. Res. 18: 4291–4302. 10.1158/1078-0432.CCR-12-0373 PMC3479663 22723371

[19] Kemp CJ (2005) Multistep skin cancer in mice as a model to study the evolution of cancer cells. Semin. Cancer. Biol. 15: 460–473. 10.1016/j.semcancer.2005.06.003 16039870

[20] Abel EL, Angel JM, Kiguchi K, DiGiovanni J (2009) Multi-stage chemical carcinogenesis in mouse skin: fundamentals and applications. Nat. Protoc. 4: 1350–1362. 10.1038/nprot.2009.120 PMC3213400 19713956

[21] Wilker E, Lu J, Rho O, Carbajal S, Beltrán L, et al (2005) Role of PI3K/Akt signaling in insulin-like growth factor-1 (IGF-1) skin tumor promotion. Mol. Carcinog. 44: 137–145. 10.1002/mc.20132 16086373

[22] Amornphimoltham P, Leelahavanichkul K, Molinolo A, Patel V, Gutkind JS (2008) Inhibition of Mammalian target of rapamycin by rapamycin causes the regression of carcinogen-induced skin tumor lesions. Clin. Cancer. Res. 14: 8094–8101. 10.1158/1078-0432.CCR-08-0703 PMC3407681 19073969

[23] Brown K, Strathdee D, Bryson S, Lambie W, Balmain A (1998) The malignant capacity of skin tumours induced by expression of a mutant H-ras transgene depends on the cell type targeted. Curr. Biol. 8: 516–524. 10.1016/s0960-9822(98)70203-9 9560338

[24] Kemp CJ, Donehower LA, Bradley A, Balmain A (1993) Reduction of p53 gene dosage does not increase initiation or promotion but enhances malignant progression of chemically induced skin tumors. Cell. 74: 813–822. 10.1016/0092-8674(93)90461-x 8374952

[25] Glick AB, Lee MM, Darwiche N, Kulkarni AB, Karlsson S, et al (1994) Targeted deletion of the TGF-beta 1 gene causes rapid progression to squamous cell carcinoma. Genes. Dev. 8: 2429–2440. 10.1101/gad.8.20.2429 7958907

[26] Han G, Lu SL, Li AG, He W, Corless CL, et al (2005) Distinct mechanisms of TGF-beta1-mediated epithelial-to-mesenchymal transition and metastasis during skin carcinogenesis. J. Clin. Invest. 115: 1714–1723. 10.1172/JCI24399 PMC1142114 15937546

[27] Matsumoto T, Jiang J, Kiguchi K, Ruffino L, Carbajal S, et al (2003) Targeted expression of c-Src in epidermal basal cells leads to enhanced skin tumor promotion, malignant progression, and metastasis. Cancer. Res. 63: 4819–4828. 12941801

[28] Chan KS, Sano S, Kiguchi K, Anders J, Komazawa N, et al (2004) Disruption of Stat3 reveals a critical role in both the initiation and the promotion stages of epithelial carcinogenesis. J. Clin. Invest. 114: 720–728. 10.1172/JCI21032 PMC514583 15343391

[29] Rundhaug JE, Pavone A, Kim E, Fischer SM (2007) The effect of cyclooxygenase-2 overexpression on skin carcinogenesis is context dependent. Mol. Carcinog. 46: 981–992. 10.1002/mc.20340 17583568

[30] Segrelles C, Lu J, Hammann B, Santos M, Moral M, et al (2007) Deregulated activity of Akt in epithelial basal cells induces spontaneous tumors and heightened sensitivity to skin carcinogenesis. Cancer. Res. 67: 10879–10888. 10.1158/0008-5472.CAN-07-2564 18006833

[31] Rundhaug JE, Gimenez-Conti I, Stern MC, Budunova IV, Kiguchi K, et al (1997) Changes in protein expression during multistage mouse skin carcinogenesis. Mol. Carcinog. 20: 125–136. 9328443

[32] Gong S, Zheng C, Doughty ML, Losos K, Didkovsky N, et al (2003) A gene expression atlas of the central nervous system based on bacterial artificial chromosomes. Nature. 425: 917–925. 10.1038/nature02033 14586460

[33] Indra AK, Li M, Brocard J, Warot X, Bornert JM, et al (2000) Targeted somatic mutagenesis in mouse epidermis. Horm. Res. 54: 296–300. 10.1159/000053275 11595821

[34] Darwiche N, Ryscavage A, Perez-Lorenzo R, Wright L, Bae DS, et al (2007) Expression profile of skin papillomas with high cancer risk displays a unique genetic signature that clusters with squamous cell carcinomas and predicts risk for malignant conversion. Oncogene. 26: 6885–6895. 10.1038/sj.onc.1210491 17525749

[35] Morris RJ, Liu Y, Marles L, Yang Z, Trempus C, et al (2004) Capturing and profiling adult hair follicle stem cells. Nat. Biotechnol. 22: 411–417. 10.1038/nbt950 15024388

[36] Barker N, Tan S, Clevers H (2013) Lgr proteins in epithelial stem cell biology. Development. 140: 2484–2494. 10.1242/dev.083113 23715542

[37] Ariki R, Morikawa S, Mabuchi Y, Suzuki S, Nakatake M, et al (2014) Homeodomain transcription factor meis1 is a critical regulator of adult bone marrow hematopiesis. Plos one. 9: e87646. 10.1371/journal.pone.0087646 PMC3911998 24498346

[38] Niessen MT, Scott J, Zielinski JG, Vorhagen S, Sotiropoulou PA, et al (2013) aPKCλ controls epidermal homeostasis and stem cell fate through regulation of division orientation. J. Cell. Biol. 202: 887–900. 10.1083/jcb.201307001 PMC3776350 24019538

[39] Estrach S, Ambler CA, Lo Celso C, Hozumi K, Watt FM (2006) Jagged 1 is a beta-catenin target gene required for ectopic hair follicle formation in adult epidermis. Development. 133: 4427–4438. 10.1242/dev.02644 17035290

[40] Rhee H, Polak L, Fuchs E (2006) Lhx2 maintains stem cell character in hair follicles. Science. 312: 1946–1949. 10.1126/science.1128004 PMC2405918 16809539

[41] Sen GL, Reuter JA, Webster DE, Zhu L, Khavari PA (2010) DNMT1 maintains progenitor function in self-renewing somatic tissue. Nature. 463: 563–567. 10.1038/nature08683 PMC3050546 20081831

[42] Wakabayashi Y, Mao JH, Brown K, Girardi M, Balmain A (2007) Promotion of Hras-induced squamous carcinomas by a polymorphic variant of the Patched gene in FVB mice. Nature. 445: 761–765. 10.1038/nature05489 17230190

[43] Malanchi I, Peinado H, Kassen D, Hussenet T, Metzger D, et al (2008) Cutaneous cancer stem cell maintenance is dependent on beta-catenin signalling. Nature. 452: 650–653. 10.1038/nature06835 18385740

[44] Beck B, Driessens G, Goossens S, Youssef KK, Kuchnio A, et al (2011) A vascular niche and a VEGF-Nrp1 loop regulate the initiation and stemness of skin tumours. Nature. 478: 399–403. 10.1038/nature10525 22012397

[45] Yuspa SH, Długosz AA, Cheng CK, Denning MF, Tennenbaum T, et al (1994) Role of oncogenes and tumor suppressor genes in multistage carcinogenesis. J. Invest. Dermatol. 103: 90–95. 10.1111/1523-1747.ep12399255 7963691

[46] Morris RJ, Fischer SM, Slaga TJ (1986) Evidence that a slowly cycling subpopulation of adult murine epidermal cells retains carcinogen. Cancer Res. 46, 3061–3066. 3698024

[47] Morris RJ, Coulter K, Tryson K, Steinberg SR (1997) Evidence that cutaneous carcinogen-initiated epithelial cells from mice are quiescent rather than actively cycling. Cancer Res. 57: 3436–3443. 9270010

[48] Vasioukhin V, Degenstein L, Wise B, Fuchs E (1999) The magical touch: genome targeting in epidermal stem cells induced by tamoxifen application to mouse skin. Proc. Natl. Acad. Sci. USA. 96: 8551–8556. 10.1073/pnas.96.15.8551 PMC17554 10411913

[49] Trapnell C, Williams BA, Pertea G, Mortazavi A, Kwan G, et al (2010) Transcript assembly and quantification by RNA-Seq reveals unannotated transcripts and isoform switching during cell differentiation. Nature. Biotechnology. 28: 511–515. 10.1038/nbt.1621 PMC3146043 20436464

[50] Kim D, Pertea G, Trapnell C, Pimentel H, Kelley R, et al (2011) TopHat2: accurate alignment of transcriptomes in the presence of insertions, deletions and gene fusions. Genome. Biology. 14: R36. 10.1186/gb-2013-14-4-r36 PMC4053844 23618408

[51] Okumura K, Sato M, Saito M, Miura I, Wakana S, et al (2012) Independent genetic control of early and late stages of chemically induced skin tumors in a cross of a Japanese wild-derived inbred mouse strain, MSM/Ms. Carcinogenesis. 33: 2260–2268. 10.1093/carcin/bgs250 22843548

