# Supplementary figures and images for: Meis1 Regulates Epidermal Stem Cells and Is Required for Skin Tumorigenesis
Source: PLoS One. 2014 Jul 11;9(7):e102111. doi: 10.1371/journal.pone.0102111 (PMC4094504; doi:10.1371/journal.pone.0102111)

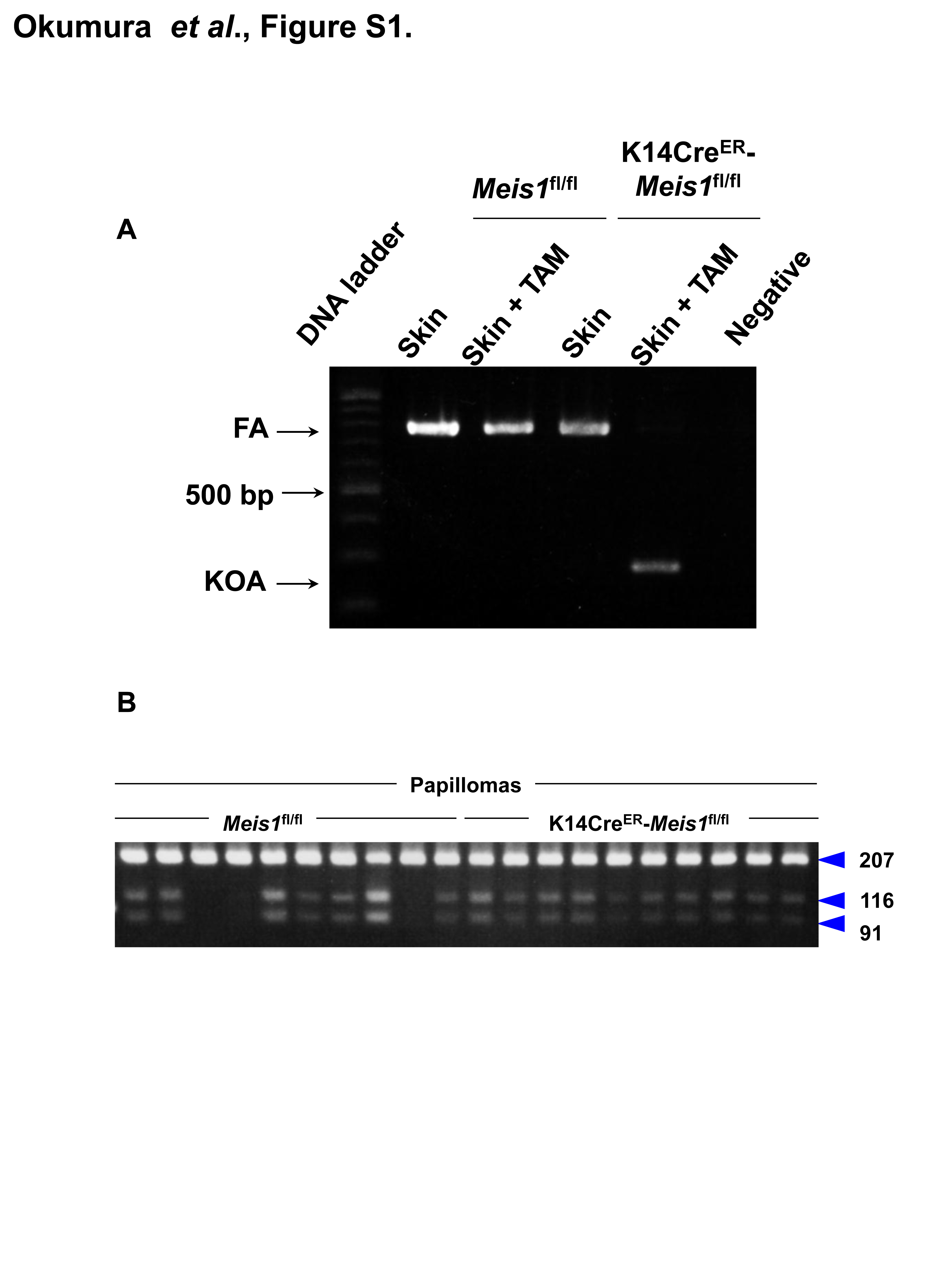

Supplement: Figure S1 — Meis1 deletion and Hras mutation analysis. (A) Confirmation of Meis1 deletion in K14CreER-Meis1fl/fl mice. Skin DNA prepared from K14CreER-Meis1fl/fl mice that were either treated (+) or untreated (−) with TAM (tamoxifen) was subjected to PCR analysis using primer pairs described elsewhere [37]. DNA from Meis1fl/fl mice was used as a control. FA denotes floxed allele. KOA denotes knockout allele. (B) XbaI digests of PCR products in the Hras gene. XbaI was used to detect the point mutation at codon 61 of the Hras gene induced by DMBA. After amplification of Hras exon 2 as previously described [51], PCR products from the mutant Hras allele produce 116- and 91-bp fragments after XbaI single digestion. (TIF) [file pone.0102111.s001.tif]

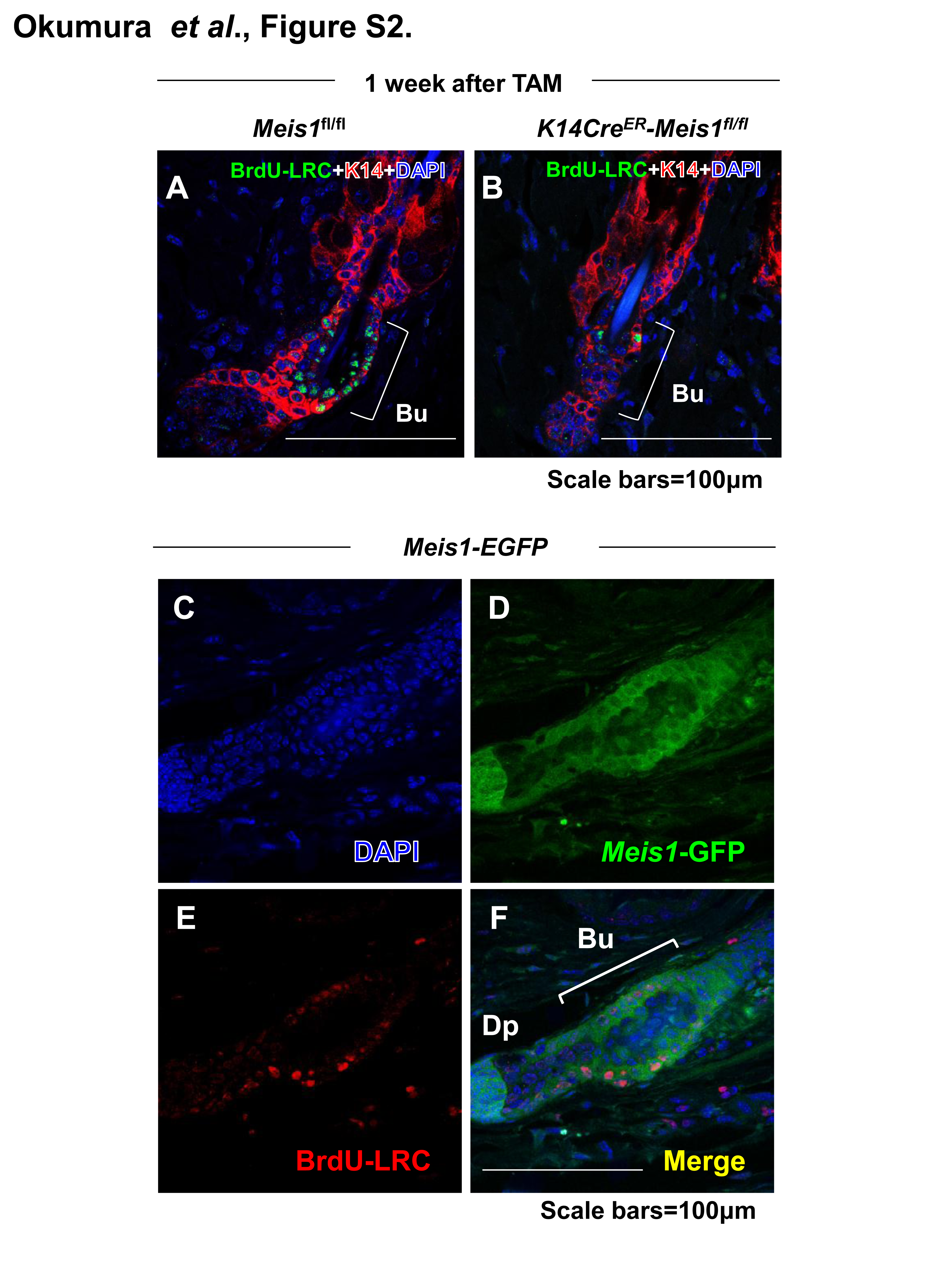

Supplement: Figure S2 — Disruption of Meis1 decreased BrdU-LRCs in the bulge. (A, B) Representative double immunostaining pattern of BrdU-LRC (green) and K14 (red) in the skin from control Meis1fl/fl mice (A) and K14CreER-Meis1fl/fl mice (B) one week after TAM treatment. Cells were counterstained with DAPI (blue). For chase experiments, BrdU was administered by peritoneal injection. See material and methods for more details of the BrdU chase experiments. (C–F) Immunofluorescence analysis to localize Meis1-EGFP-positive cells in adult mouse skin. Dorsal back skin sections from 8-week-old Meis1-EGFP reporter mice were stained with anti-GFP antibody, in combination with anti-BrdU antibody. Cells were counterstained with DAPI. (C–E) DAPI (blue), Meis1-EGFP (green) fluorescence, and BrdU-LRC (red) are shown. (F) The merged image of Meis1-EGFP with BrdU-LRC. Abbreviations: “Bu” means “bulge” and “Dp” means “dermal papilla”. Scale bars, 100 µm. (TIF) [file pone.0102111.s002.tif]

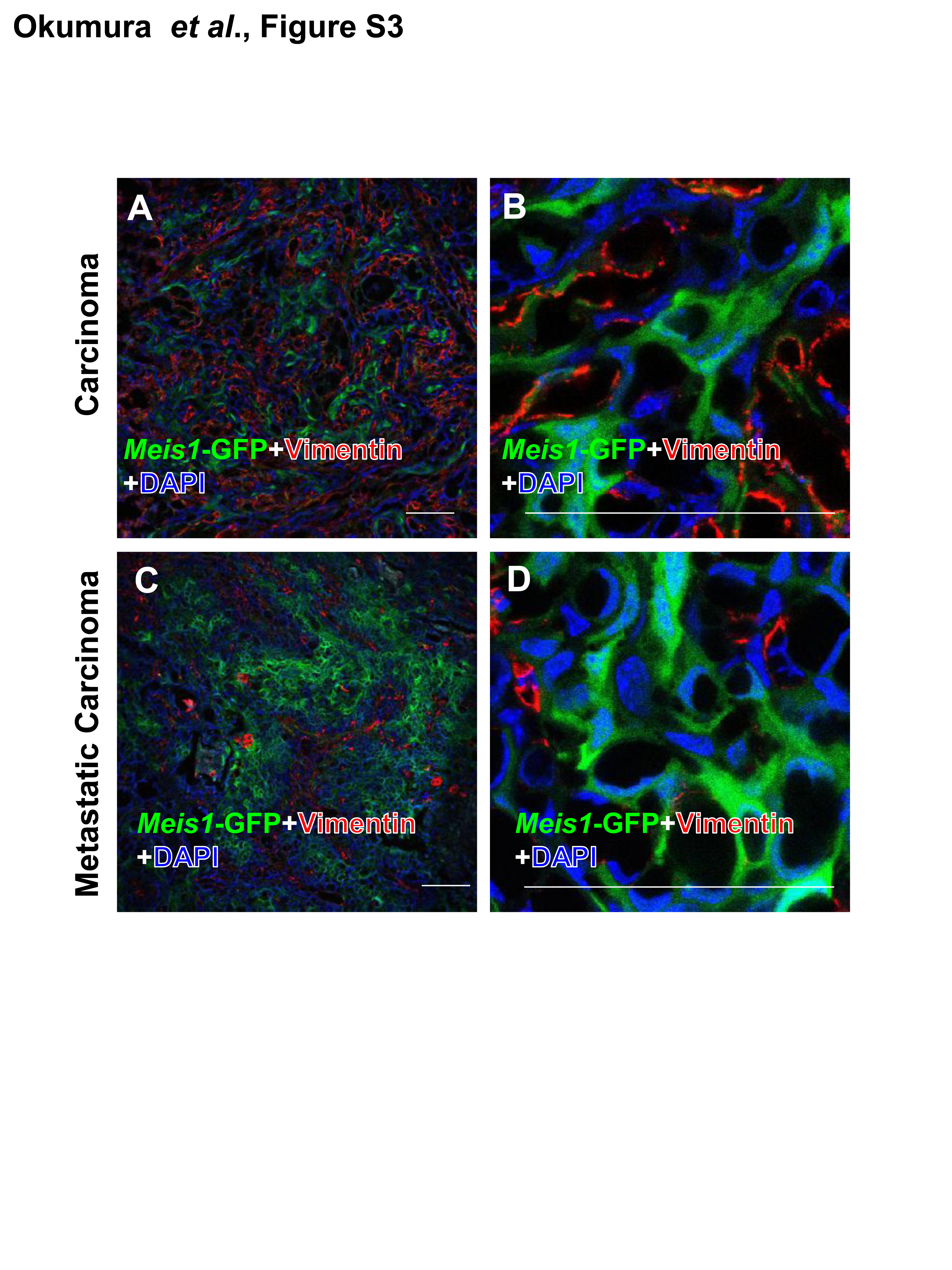

Supplement: Figure S3 — Expression of Meis1 did not overlap with vimentin in malignant tumors. Immunofluorescence microscopic analysis of papillomas, carcinomas in situ, and metastatic carcinomas induced by DMBA/TPA in Meis1-EGFP reporter mice. (A–D) The sections were stained with anti-GFP antibody (green), in combination with anti-vimentin antibody (red). Cells were counterstained with DAPI. (A) A carcinoma in situ at 40 weeks after initiation. (B) A magnified image of (A). (C) A metastatic carcinoma at 40 weeks after initiation. (D) A magnified image of (C). Scale bars, 100 µm (A, C). Scale bars, 50 µm (B, D). (TIF) [file pone.0102111.s003.tif]
